# ASO Author Reflections: Synoptic Reporting for Melanoma Nodal Surveillance Ultrasonography

**DOI:** 10.1245/s10434-024-15735-6

**Published:** 2024-07-16

**Authors:** Kelsey B. Montgomery, Ashley M. Holder, Kristy K. Broman

**Affiliations:** 1 Department of Surgery, University of Alabama at Birmingham, Birmingham, AL USA; 2 Department of Surgical Oncology, University of Texas MD Anderson Cancer Center, Houston, TX USA; 3 Institute for Cancer Outcomes and Survivorship, University of Alabama at Birmingham, Birmingham, AL USA; 4 Birmingham Veterans Affairs Medical Center, Birmingham, AL USA

## Past

Following the Second Multicenter Selective Lymphadenectomy Trial (MSLT-II), the use of nodal surveillance in sentinel lymph node-positive (SLN+) melanoma has significantly increased, prompting the need for reliable, high-quality nodal surveillance ultrasound (U/S).^1^ However, prior studies have demonstrated inconsistent use of MSLT-II U/S criteria in real-world practice, as well as concerns from some surgical and medical oncologists about the quality of current nodal U/S studies.^2,3^ Across multiple cancer types, the development of synoptic reporting templates for screening or surveillance imaging has enabled improved communication between radiologists and oncologists, including better delineation of appropriate next steps in management.^4^

## Present

In this study, we evaluated the post-implementation outcomes of a synoptic reporting template for melanoma nodal surveillance U/S at a single tertiary cancer center.^5^ Following implementation, there was a significant increase in the number of MSLT-II criteria reported, as well as the clinically actionable recommendations reported for studies with abnormal findings. Additionally, there was high satisfaction from surgeons, surgical oncology advanced practice providers (APPs), and radiologists for the synoptic template. Notably, this process was interdisciplinary, with multiple radiologist champions who were integral to the successful implementation of the synoptic template, including engaging with radiology faculty, educating of sonography technicians, and identifying logistical issues to be addressed in real-time.

## Future

Moving forward, this synoptic template for melanoma nodal surveillance may improve the information provided to multidisciplinary team members by standardizing an imaging modality that may otherwise be somewhat user-dependent. This standardization may also produce opportunities for multi-institutional research with a common reporting language across participating sites, or for potential dissemination to smaller centers that may be more easily accessible to patients locally. Future work will aim to evaluate the feasibility of this synoptic template at other cancer centers and identify barriers and facilitators to successful implementation in these new settings.
